# Supplementary material for: Ethosomes and Transethosomes as Cutaneous Delivery Systems for Quercetin: A Preliminary Study on Melanoma Cells
Source: Pharmaceutics. 2022 May 11;14(5):1038. doi: 10.3390/pharmaceutics14051038 (PMC9147749; doi:10.3390/pharmaceutics14051038)
Supplement: Supplementary file 1 [file pharmaceutics-14-01038-s001.zip › pharmaceutics-1705506-supplementary.pdf]

Supplementary materials

Ethosomes and transethosomes as cutaneous delivery systems for quercetin: a preliminary study on melanoma cells

Francesca Ferrara, Mascia Benedusi, Maddalena Sguizzato, Rita Cortesi, Anna Baldisserotto, Raissa Buzzi, Giuseppe Valacchi*, and Elisabetta Esposito*

Table S1. Membranes employed for in vitro release (IVRT) and permeation (IVPT) tests

	IVRT		IVPT	
**Characteristics**	**NY**	**PTFE**	**STRAT-M®**	**SCE**
composition	nylon (polyamide)	polytetra -fluoroethylene	polyethersulfone -polyolefin	stratum corneum-epidermis
character	hydrophilic	lipophilic	amphiphilic	amphiphilic
pore size (μm)	0.22	0.22	n.a.	n.a.
thickness (μm)	150–187[a]	156[a]	317[a]	22[b]

n.a.: not available; a: as indicated by the supplier; b: as measured by SEM

Table S2: Mathematical parameters describing QT release evaluated from IVRT data.

Formulation code	Order of process	c	m	R^2
$ETO_{0.9}$-QT [N]	Zero order	-0.3080	0.0099	0.9948
	First order	-0.2900	0.1810	0.9162
	Higuchi	-1.2760	2.6513	0.9369
T-$ETO_{0.9}$-QT [N]	Zero order	-0.3485	0.4885	0.9936
	First order	-0.8549	0.2210	0.8751
	Higuchi	-1.6367	0.8651	0.9271
T-$ETO_{2.7}$-QT [N]	Zero order	-0.8961	1.1840	0.9834
	First order	-0.7301	0.2670	0.8883
	Higuchi	-1.8371	3.0182	0.8900
$ETO_{0.9}$-QT [P]	Zero order	0.5911	0.0123	0.9877
	First order	0.2680	0.0130	0.9299
	Higuchi	-1.1290	3.3682	0.9715
T-$ETO_{0.9}$-QT [P]	Zero order	1.4880	0.0131	0.9846
	First order	0.5024	0.0764	0.9491
	Higuchi	-0.5341	4.0566	0.9814
T-$ETO_{2.7}$-QT [P]	Zero order	1.0000	1.6167	0.9809
	First order	0.3969	0.1033	0.8904
	Higuchi	-1.3650	4.8913	0.9760

N: Nylon membrane: P: PTFE membrane

Table S3: IVPT parameters of the indicated forms determined by Franz cell associated to STRAT-M®

Formulation code	Jss[1] ($\mu g\ cm^{-2}\ h^{-1}$)	T_{lag}[2] (h)	Kp[3] ($cm\ h^{-1}\ 10^{-3}$)	D[4] ($cm\ h^{-1}$) x 10-	P[5] membrane/vehicle	A_{QT}[6] ($\mu g\ cm^{-2}$]	M_{QT}[7] ($\mu g\ cm^{-2}$)
$ETO_{0.9}$-QT	0.09 ± 0.01	3.01 ± 0.21	0.18 ± 0.01	5.0 ± 0.2	11.41 ± 1.21	3.0 ± 0.2	15 ± 1.0
T-$ETO_{0.9}$-QT	0.45 ± 0.05	2.82 ± 0.11	0.90 ± 0.05	6.0 ± 0.2	47.55 ± 2.00	4.8 ± 1.2	18 ± 2.0
T-$ETO_{2.7}$-QT	0.05 ± 0.01	3.21 ± 0.20	0.10 ± 0.01	5.2 ± 0.3	6.09 ± 0.42	2.9 ± 0.9	20 ± 2.5
SOL-QT	0.33 ± 0.09	2.10 ± 0.21	0.66 ± 0.09	8.0 ± 0.1	26.15 ± 3.43	2.4 ± 1.0	12 ± 1.5

1: steady state flux per unit area, 2: lag time; 3: permeability coefficient; 4: diffusion coefficient; 5: partition coefficient; 6: cumulative amount of QT diffused at 24 h; 7: QT associated to the membrane after 24 h; data are the mean of 12 independent Franz cell experiments ± s.d..

Table S4: IVPT parameters of the indicated forms determined by Franz cell associated to SCE

Formulation code	Jss[1] ($\mu g\ cm^{-2}\ h^{-1}$)	T_{lag}[2] (h)	Kp[3] ($cm\ h^{-1}\ 10^{-3}$)	A_{QT}[4] ($\mu g\ cm^{-2}$)	M_{QT}[5] ($\mu g\ cm^{-2}$)
$ETO_{0.9}$-QT	0.60 ± 0.08	1.90 ± 0.10	1.20 ± 0.08	4.8 ± 0.5	24± 1.5
T-$ETO_{0.9}$-QT	1.20 ± 0.10	1.15 ± 0.11	2.40 ± 0.10	10.0 ± 1.2	30± 2.0
T-$ETO_{2.7}$-QT	1.09 ± 0.01	2.10 ± 0.22	2.18 ± 0.01	7.0 ± 0.7	28 ± 3.2
SOL-QT	0.19 ± 0.15	1.13 ± 0.51	0.38 ± 0.20	1.6 ± 0.2	16 ± 3.5

1: steady state flux per unit area, 2: lag time; 3: permeability coefficient; 4: cumulative amount of QT diffused at 24 h; 7: QT associated to the membrane after 24 h; data are the mean of 12 independent Franz cell experiments ± s.d..

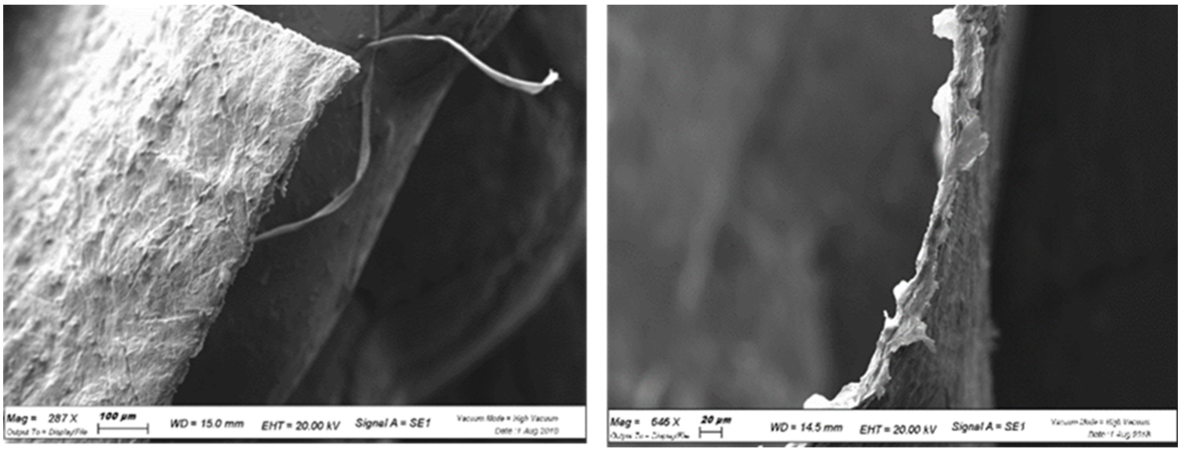

(a) (b)

Figure S1. SEM images of human SCE membranes employed for IVPT. SCE samples were prepared as reported in section 2.7.

(a)

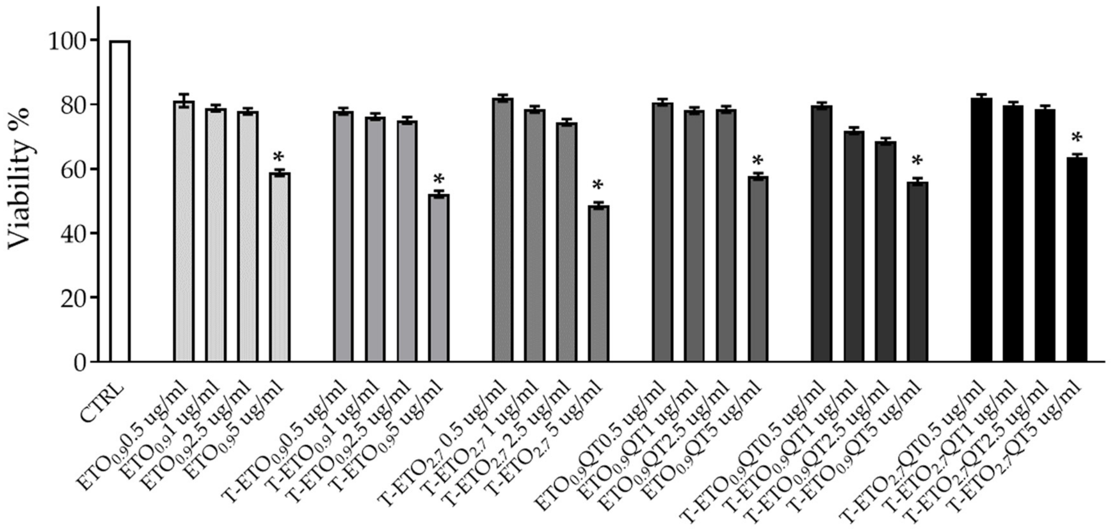

(b)

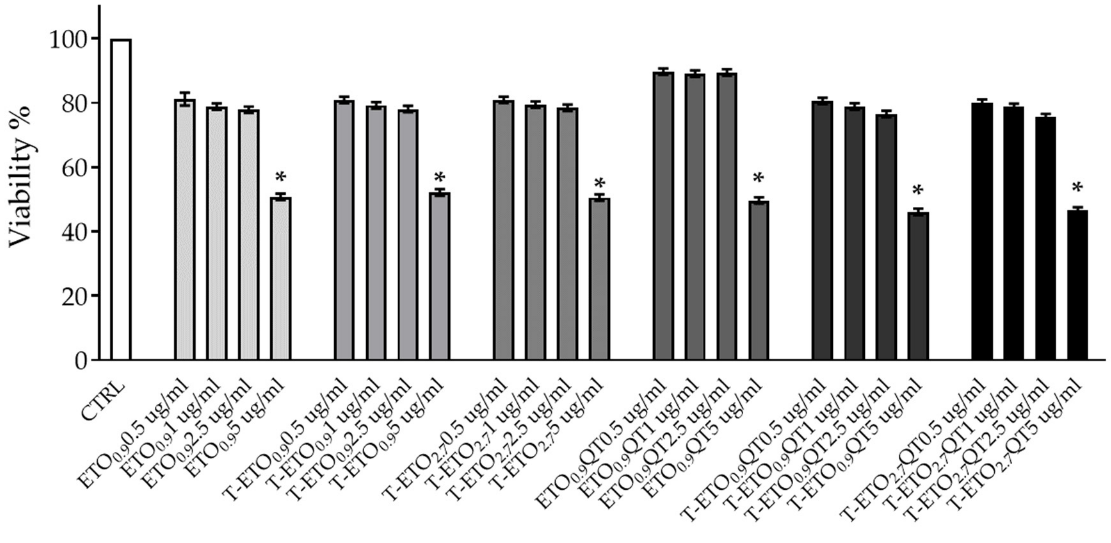

Figure S2. HaCaT cell (a) and HT-144 melanoma cells (b) viability evaluated by MTT test after 24 h of treatment with QT-loaded or unloaded $ETO_{0.9}$, T-$ETO_{0.9}$ and T-$ETO_{2.7}$. Data are the results of three independent experiments performed in triplicate. Data appear as the mean±SE. * $p < 0.05$ by two-way ANOVA (Post hoc test: Tukey Test) Ctrl vs treated cells.

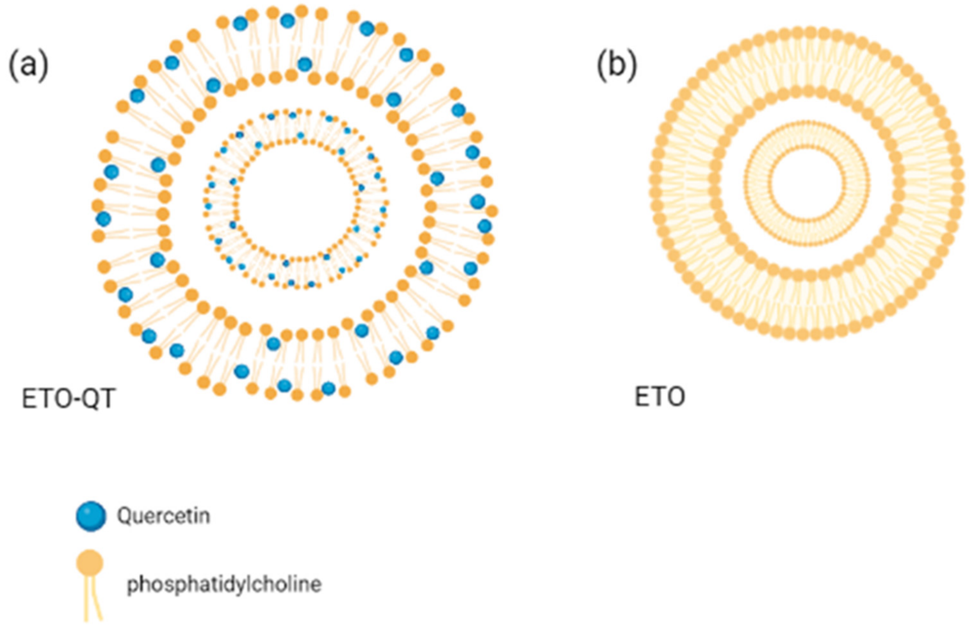

Figure S3: Graphical representation showing the possible QT positioning within PC bilayers, resulting in larger vesicles in the case of loaded (a) with respect to unloaded (b) ethosomes.

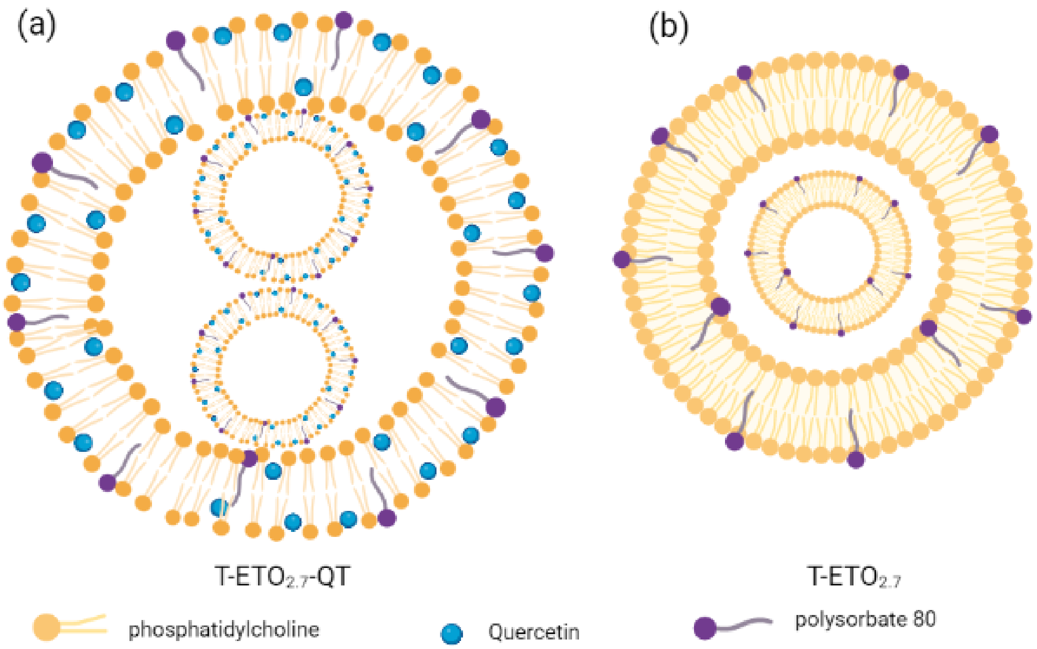

Figure S4: Graphical representation showing the possible architectural organization of T-ETO$_{2.7}$ (a). The presence of polysorbate 80 could stabilize the vesicles, possibly improving QT interaction with PC in T-ETO$_{2.7}$-QT (b).
